# Kikuchi–Fujimoto Disease: A Case Series and Review of the Literature

**DOI:** 10.3390/diseases12110271

**Published:** 2024-11-01

**Authors:** Arunima Deb, Vielka Fernandez, Ekim Kilinc, Hisham F. Bahmad, Nicholas S. Camps, Vathany Sriganeshan, Ana Maria Medina

**Affiliations:** 1 The Arkadi M. Rywlin M.D. Department of Pathology and Laboratory Medicine, Mount Sinai Medical Center, Miami Beach, FL 33140, USA; arunima.deb@msmc.com (A.D.); vielka.fernandez@msmc.com (V.F.); ekim.kilinc@msmc.com (E.K.); vathany.sriganeshan@msmc.com (V.S.); ana.medina@msmc.com (A.M.M.); 2 Department of Internal Medicine, Mount Sinai Medical Center, Miami Beach, FL 33140, USA; nicholas.camps@msmc.com; 3 Department of Pathology, Herbert Wertheim College of Medicine, Florida International University, Miami, FL 33199, USA

**Keywords:** Kikuchi–Fujimoto, histiocytic necrotizing lymphadenopathy, EBV, HIV, case series

## Abstract

Kikuchi–Fujimoto disease (KFD), also known as histiocytic necrotizing lymphadenitis, is a rare, self-limiting disorder characterized by fever typically lasting for 1 week up to 1 month and painful necrotizing lymphadenopathy, primarily affecting young adults of Asian ancestry. Although the exact cause remains unclear, infectious and autoimmune mechanisms have been implicated in the pathogenesis of the disease. In this case series, we aim to describe the histopathological features of KFD over a ten-year period at Mount Sinai Medical Center of Florida, and review the current understanding of its pathogenesis, clinical presentation, diagnosis, and management. A retrospective review of our pathology database between January 2013 and May 2024 was performed to identify patients diagnosed with KFD at our institution. Eight cases of KFD were identified, with a mean age of 35 years (range 24–49) and slight male predilection (5:3). Three patients exhibited leukopenia, and two had concurrent HIV infection. One patient developed systemic lupus erythematosus (SLE), and another developed IgA nephropathy during follow-up. Histopathological examination revealed the characteristic features of KFD, including lymph node architectural effacement, histiocytic infiltration, and necrosis. In conclusion, KFD remains a diagnostic challenge due to its overlapping clinical features with other infectious and autoimmune diseases, particularly SLE. While most cases resolve spontaneously, long-term follow-up is warranted due to the potential for recurrence and autoimmune associations.

## 1. Introduction

Kikuchi–Fujimoto disease (KFD), also known as histiocytic necrotizing lymphadenitis, is a rare disorder characterized by fever and subacute necrotizing regional lymphadenopathy [1]. It was first described in 1972 by Japanese pathologists Kikuchi and Fujimoto [2,3]. It predominantly affects women aged 20 to 35, but pediatric cases also exist, showing a varying gender distribution [4]. The cause of KFD remains unclear, but both viral infections (including Epstein–Barr virus (EBV), herpes simplex virus (HSV), varicella zoster virus (VZV), human herpesviruses (HHV) 6, 7, and 8, parvovirus B19, paramyxovirus, parainfluenza virus, rubella, cytomegalovirus (CMV), hepatitis B virus, human immunodeficiency virus (HIV), human T-lymphotropic virus type 1 (HTLV-1), and dengue virus) and autoimmune mechanisms have been suggested as potential triggers [5]. Other infectious agents reported include *Brucella*, *Bartonella henselae*, *Yersinia enterocolitica*, *Toxoplasma gondii*, *Entamoeba histolytica*, and *Mycobacterium szulgai* [1,6,7]. The body’s immune response postulated to be implicated in KFD is cell-mediated in response to a variety of antigens in genetically susceptible people [5].

Patients with KFD are found to have human leukocyte antigen (HLA) class II alleles, specifically HLA-DPA1 and HLA-DPB1, which are more prevalent in Asians compared to the general world population [8]. This explains the association between KFD and a number of systemic autoimmune diseases, such as systemic lupus erythematosus (SLE), Wegener granulomatosis, Sjögren syndrome, and Graves disease, among others [6,9]. Interestingly, studies using electron microscopy have shown that within the cytoplasm of lymphocytes and histiocytes in lymph nodes of KFD patients are tubular reticular structures. Those structures are similar to the ones present in endothelial cells and lymphocytes of patients with SLE [10].

KFD presents with non-specific symptoms such as fever, fatigue, and painful lymphadenopathy [11]. Skin involvement occurs in 30–40% of cases, mainly in children, presenting as erythematous papules or macules [12,13]. Central nervous system involvement is rare but can manifest as aseptic meningitis or optic neuritis [14]. Diagnosis relies on histopathological examination, with distinct subtypes evolving over the course of the disease [15,16,17]. Early treatment involves analgesia, antipyretics, and NSAIDs [18]. Corticosteroids are reserved for severe cases or extranodal involvement [19]. Excisional biopsy of lymph nodes can be both diagnostic and therapeutic. Clinical differential diagnosis includes hematologic malignancies, including classic Hodgkin and non-B-cell and T-cell Hodgkin lymphomas, infectious diseases such as tuberculosis, histoplasmosis, leprosy, syphilis, cat-scratch disease, infectious mononucleosis, HSV lymphadenitis, and connective tissue diseases like SLE [20,21,22]. Histopathological findings and specific diagnostic tests help distinguish KFD from these conditions.

Treatment is generally supportive, and most cases resolve within 1 to 2 months. Recurrence rates are around 5%, and long-term follow-up is essential due to the risk of developing autoimmune diseases, particularly SLE.

## 2. Materials and Methods

### 2.1. Study Design, Setting, and Objectives

The pathological database of surgical specimens at Mount Sinai Medical Center (Miami Beach, FL, USA) was reviewed, searching for patients diagnosed with KFD during a ten-year period, from 1 January 2013 to 31 May 2024. This study represents a case series analysis with no direct physical risk to the patients in the study. Chart review was performed, and patient information was collected.

### 2.2. Ethical Considerations

Approval of the Institutional Review Board (IRB) of Mount Sinai Medical Center of Florida was granted prior to commencement of this study. All protocols followed in our retrospective cohort study were performed in accordance with guidelines and regulations of The Code of Ethics of the World Medical Association (Declaration of Helsinki). This study was performed in a manner that ensures confidentiality of patients.

### 2.3. Patients’ Selection

Inclusion criteria included patients who underwent excisional or core biopsies from lymph nodes during the time-period specified. No exclusion criteria were present.

### 2.4. Clinicopathological Parameters of Patients

Clinical and pathological parameters of patients were retrospectively retrieved from electronic medical records, including age, clinical data (signs and symptoms, imaging results), type of surgery, risk factors, pathological diagnosis, and follow-up data.

## 3. Results

While performing a 10-year retrospective search of our institution database, we found eight cases of KFD. The age of the patients ranged from 24 to 49 years at the time of diagnosis, with a mean age of 35 years. The clinical and demographic characteristics of the patients are summarized in Table 1. Slight male predilection was noticed (M:F = 5:3). Out of the eight patients, three were African American, one was Hispanic, two were Asian, and one was Caucasian. The demographic and clinical data of one patient were unavailable on the electronic medical record database. The most common clinical presentation was fever and night sweats. All the patients were found to have lymphadenopathy. In one patient, the presenting complaint was a groin mass, and in another, it was generalized lymphadenopathy. Bilateral axillary lymphadenopathy was the chief complaint in one patient. On further investigation, she was found to have metastatic breast carcinoma to the axilla and KFD was an incidental finding. One female patient developed erythematous macules on the face during the course of the disease. All the other patients presented with cervical lymphadenopathy.

Three patients presented with leukopenia. One patient was lost to follow up. Two of the patients had been diagnosed with HIV. One patient (Patient 1, Table 1) subsequently developed arthralgia and tested positive for anti-nuclear antibody (ANA) and anti-double stranded deoxyribonucleic acid antibody (anti-dsDNA). He was diagnosed with SLE. Another patient developed IgA nephropathy.

**Figure 1:**
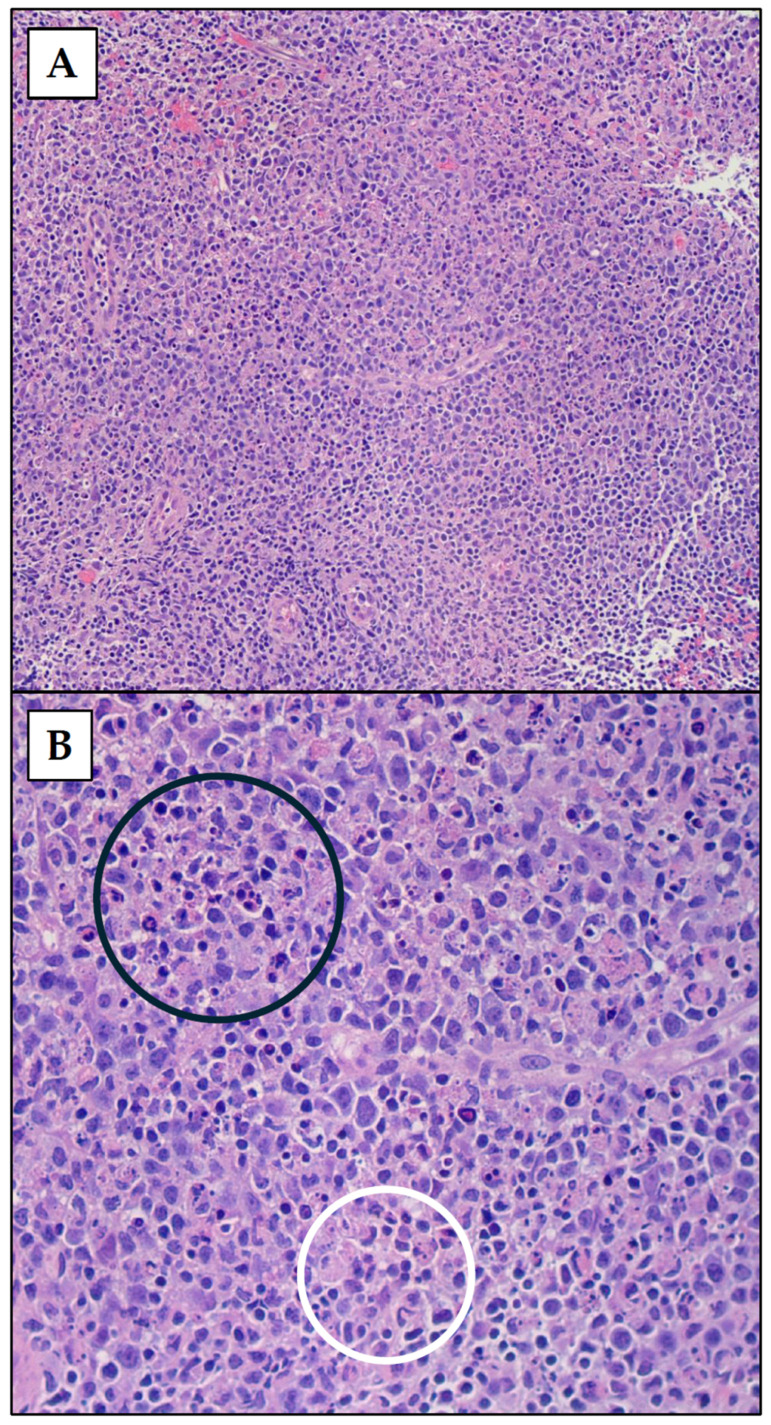
Hematoxylin and eosin (H&E)-stained sections of a lymph node showing karyorrhectic debris (black circle) with histiocytes showing crescentic nuclei (white circle). Microscopic images were examined at 100× (**A**) and 200× (**B**) objectives.

Excisional biopsies of the affected lymph nodes were performed in five cases and core biopsies in three cases. Seven cases showed architectural effacement of the lymph nodes, with paracortical expansion by numerous histiocytes with crescentic nuclei, immunoblasts in varying proportions and necrosis with abundant nuclear debris (Figure 1). Neutrophils were not identified. The histiocytes stained positively for CD68, CD163, CD4, and myeloperoxidase (MPO). In one case, paracortical and follicular hyperplasia with a focus of a highly atypical lymphoid proliferation that stained positively for histiocytic markers and MPO was the predominant finding.

## 4. Discussion

### 4.1. History of Kikuchi–Fujimoto

Kikuchi–Fujimoto Disease (KFD) is a rare disorder that is characterized mainly by fever and prolonged lymphadenopathy. It is a benign and self-limited condition that was first described by Japanese pathologists Kikuchi and Fujimoto in 1972 [2,3]. Kikuchi and Fujimoto independently described two separate cases, one presenting with ‘lymphadenitis showing focal reticulum cell hyperplasia, nuclear debris, and phagocytosis’ and another revealing ‘subacute necrotizing cervical lymphadenitis’, hence the name ‘Kikuchi–Fujimoto disease’ [2,3].

### 4.2. Epidemiology

KFD is uncommon in the United States, with most cases occurring in Asia, although there have been cases described elsewhere in the world in the literature. Women aged 20 to 35 years have a higher incidence of KFD [4], but pediatric cases have also been described. In a Korean report of 20 patients younger than 18 years, the sex distribution was equal [23]. Other studies suggested boys are slightly more frequently affected than girls among children, in contrast to older patients. The clinical features of KFD may differ according to age and sex. In addition, KFD has a reported recurrence rate of 3–4% [24] but pediatric studies have shown a higher recurrence rate of up to 42.4% [25,26]. In our study, there was a slight male predilection.

### 4.3. Risk Factors

Due to its low incidence and rather non-specific clinical features, KFD has not been well understood. Although infectious agents and T-cell-mediated autoimmune response have been hypothesized as the etiology, the pathogenesis of KFD remains unclear [1]. KFD shares many clinical and histopathologic features of a viral infection [5]. In KFD cases, several viruses have been documented: most frequently DNA viruses including EBV, HHV 6, 7, and 8, parvovirus B19, HSV types 1 and 2, VZV, CMV, human papillomavirus (HPV), and hepatitis B virus. The RNA viruses isolated include dengue virus, rubella virus, paramyxovirus, parainfluenza virus, and retroviruses [1,4,24,27,28,29,30]. Although EBV has been described in some studies as one of the most common viral infections associated with KFD, immunohistochemistry detected EBV-encoded protein in only 1 out of 10 patients showing EBV by in situ hybridization [27,31,32,33].

Autoimmune mechanisms have also been suggested as a possible driver for KFD, with clinical and pathological features mimicking Sjögren syndrome and SLE [33,34]. Specifically, KFD shares age and gender predisposition as well as histologic features with SLE. Therefore, KFD is thought to be a self-limiting SLE-like autoimmune disorder caused by virus-infected transformed lymphocytes [10]. The increased risk of developing autoimmune syndromes (such as Hashimoto thyroiditis, primary Sjögren syndrome, antiphospholipid syndrome, and leukocytoclastic vasculitis), particularly in females, supports the hypothesis of the autoimmune mechanism [35,36].

KFD has also been linked to coronavirus disease and the COVID-19 vaccination [35,36,37,38,39,40,41]. As most patients recovered quickly and completely, whether the association is causal or not remains unclear.

### 4.4. Clinical Presentation

Symptoms in KFD can present acutely or evolve over a period of 2–3 weeks. In a series of 244 cases, non-specific symptoms like fever early on presented in approximately 35% of the patients, followed later by cutaneous involvement, arthritis, fatigue and hepatosplenomegaly [11]. The fever associated with KFD is generally intermittent and low grade and occurs in up to 50% of cases [42]. Other constitutional symptoms like anorexia, weight loss, night sweats, and chills were observed in approximately 86% of the cases in a retrospective study of 91 cases [43]. These symptoms are more frequently associated with extranodal involvement [44].

The most common clinical feature is lymphadenopathy, which is an essential criterion in the diagnosis and is present in virtually all cases [11]. Lymph node enlargement can vary from 1 to 2 cm in diameter, while there have been cases with a reported diameter of up to 7 cm [16]. The lymphadenopathy is often painful but may also present as a dull discomfort. Unilateral posterior cervical involvement is the most common affected site [16,42]. Generalized lymphadenopathy is present in approximately 1–20% of cases and sites like mediastinal, epitrochlear, mesenteric, axillary, iliac, celiac, intraparotid, inguinal, retroperitoneal, and peripancreatic nodes have been reported [4]. In cases with mesenteric involvement, the disease could be misdiagnosed as acute appendicitis [45].

Skin is the second most common organ involved, occurring in 30–40% of cases [12,13]. A diverse range of cutaneous presentations have been described, like lichen planus, alopecia, scales, and leukocytoclastic vasculitis [31,46]. Most lesions are non-specific, like erythematous papules or macules [47]. The presence of a malar rash, similar to the one observed in SLE, can also occur [13]. Mucosal involvement can manifest as ulcers, oropharyngeal erythema and conjunctival injection [48].

Involvement of the central nervous system has been described, usually presenting as a spectrum of entities like aseptic meningitis, optic neuritis, meningoencephalitis, and acute cerebellar manifestations [14]. Of these, aseptic meningitis is the most common neurological complication.

### 4.5. Diagnosis

The most common laboratory abnormalities associated with KFD are leukopenia in approximately 18% of cases, increased erythrocyte sedimentation rate in 16%, and anemia of chronic disease in 9% [11]. Atypical lymphocytes can be present, as well as mildly increased liver function tests and elevated serum lactate dehydrogenase [42,49]. Bone marrow studies are not currently recommended in KFD.

The rapid onset of lymphadenopathy seen in KFD usually leads to the use of imaging to rule out infectious processes or malignancy, awaiting a definitive diagnosis with histopathology. The presence of an echogenic hilum and posterior cervical involvement can help distinguish KFD from lymphoma during ultrasound [50]. Necrosis and internal calcifications are more frequently observed in infectious processes, like tuberculous lymphadenitis [50,51]. The lack of necrosis along with cortical attenuation can help distinguish KFD from reactive hyperplasia and tuberculous lymphadenitis [52].

### 4.6. Histopathology

Histopathological findings include partial disruption of the follicular architecture, with follicular hyperplasia and necrosis involving both the cortex and paracortex. A prominent infiltrate composed of histiocytes with crescentic nuclei, lymphocytes, and immunoblasts is present [15]. Occasional eosinophils can be found; however, the characteristic feature is the absence of segmented neutrophils [53]. Infiltration of the capsule can occur, and karyorrhectic nuclear debris can be irregularly dispersed, with a large accumulation of histiocytes at the edge of the necrotic areas. Nonetheless, coagulative necrosis by itself is not diagnostic of KFD [17].

As the disease progresses, histopathological findings evolve, leading to the appearance of three different histological subtypes or stages. The early phase is described as the proliferative type, followed by a late phase known as the necrotizing type and the xanthomatous type at the end of the clinical course. The most common one, presenting in approximately 50% of cases, is the necrotizing type, while the xanthomatous type is only seen in about 20% of cases [16]. The proliferative type is characterized by follicular hyperplasia and expansion of the paracortex by an infiltrate of lymphocytes, histiocytes, and T- and B-cell immunoblasts. Abundant immunoblasts with irregular nuclear contours may mimic lymphoma [17]. Phagocytosis of nuclear debris and the predominance of histiocytes are the hallmarks of the necrotizing type [54]. Aggregates of foamy histiocytes are characteristic of the xanthomatous type, and this subtype might be indicative of a healing phase [55]. CD8-positive T lymphocytes and CD68-positive histiocytes are identified by immunohistochemical staining [54]. Strong expression of CD123 can be observed in plasmacytoid dendritic cells, while myeloperoxidase, lysozyme, CD163, CD68, and CD4 are diffusely positive in histiocytes [56].

### 4.7. Differential Diagnosis

The non-specific symptoms of lymphadenopathy and fever seen in KFD can be misdiagnosed with malignant entities like non-Hodgkin lymphoma, infectious diseases like tuberculous lymphadenitis and cat-scratch disease, or autoimmune diseases like SLE. Similarities have been recognized between connective tissue diseases like SLE and KFD [20]. The two can be easily confused with one another because of their similar epidemiological characteristics and tendency to occur in young women [57]. Clinically, SLE may manifest as fever, fatigue, lymphadenopathy, rash, leukocytosis, urinary protein, and hepatosplenomegaly, which can be associated with necrotizing lymphadenitis. In SLE, 20% of the cases have lymph node biopsies that are histologically indistinguishable from KFD. However, SLE patients have some other typical clinical manifestations, such as butterfly erythema, discoid erythema, light hypersensitivity, and arthritis, which are generally not present in KFD. Patients with SLE also have some specific diagnostic tests, such as anti-dsDNA antibodies and anti-Sm antibodies. Although both SLE and KFD share features like proliferation of immunoblasts and plasmacytoid dendritic cells in the foci of necrosis, the presence of hematoxylin bodies and abundant plasma cells is a key finding for the histopathological diagnosis of SLE [58]. In an investigation involving 30 patients with KFD and 6 patients with SLE, it was found that CD30 immunostaining was useful in distinguishing between KFD and SLE since KFD has considerably more CD30+ cells than SLE does, and most of these cells are found surrounding necrotic areas. These CD30+ cells, which are CD8+ activated cytotoxic T cells near necrotic regions and are indicative of KFD, were most common in females with modest symptoms and normal laboratory results [59]. It must be noted that if necrotizing lymphadenitis is associated with antinuclear antibody positivity, patients must be monitored for early manifestations of SLE, as many of them will gradually develop SLE [9,60,61]. The association between KFD and SLE remains unclear and necessitates long-term follow-up [33,62].

While the fever associated with KFD is usually irregular, fever in non-Hodgkin lymphoma tends to be relapsing, and fever in tuberculous lymphadenitis is generally intermittent [21]. Most cases of KFD are self-limiting, unlike the prolonged clinical course seen in non-Hodgkin lymphoma, tuberculous lymphadenitis, and SLE. Histopathological findings are key to distinguishing KFD from these entities. The presence of monoclonal lymphocytes is diagnostic for non-Hodgkin lymphoma, while granulomatous inflammation and central necrosis, along with positive serology for *Bartonella henselae*, are diagnostic for cat-scratch disease [21]. Serological tests for EBV, HHV 6 and 8, CMV, toxoplasmosis, *Y. enterocolitica*, HIV, cat-scratch disease, and other infectious agents are often performed in a suspected case of KFD to exclude other possible causes of fever and lymphadenopathy. However, the histologic features of lymphadenitis associated with these microbial agents are clearly distinguishable from those of KFD [63,64,65].

### 4.8. Prognosis

In most patients, spontaneous resolution of KFD is noted. However, 3–21% of these patients may have recurrent disease [6,43,60]. Fatality, though rare, may result from heart failure, hemophagocytic syndrome, and pulmonary hemorrhage [66,67,68,69]. In recurrent disease, multiple nodal site involvement (in 73% of patients) and an association with autoimmune diseases (in 32% of patients) can be seen [70].

## 5. Conclusions

In summary, KFD is a rare and non-neoplastic disorder primarily affecting young adults with a female predominance. Its etiology remains unclear. It can present with fever, lymphadenopathy, and skin involvement. Treatment is generally supportive, with a need for long-term follow-up due to potential associations with autoimmune diseases like SLE or development of recurrent disease.

## Figures and Tables

**Table 1:** Clinical and demographic characteristics of the patients.

Case Number	Age (Years)	Sex	Ethnicity	Clinical Features on Presentation	Lymph Node Involvement	Other Medical Conditions	Other Lab Results
1	36	M	African American	Night sweats, fever, weight loss, and shortness of breath for 1 month	Right supraclavicular, bilateral axillary, retroperitoneal, bilateral inguinal	SLE	HCV, HHV-8, Hep-B negative No growth detected on blood bacterial, mycobacterial and viral lymph node cultures
2	35	M	African American	Left groin mass	Left inguinal	Recurrent major depressive disorder	HIV, HCV negative No growth detected on bacterial, mycobacterial and viral blood cultures
3	49	F	Hispanic	Shortness of breath	Bilateral axillary	Metastatic breast cancer	No growth detected on bacterial, mycobacterial and viral pleural fluid cultures
4	24	M	Asian	Fever and night sweats for 10 days	Left submandibular	IgA nephropathy and HIV infection	CMV negative (IHC) No growth detected on bacterial blood cultures
5	49	M	Caucasian	Fever and night sweats	Left supraclavicular	HIV infection, Hepatitis B infection, and history of tuberculosis and syphilis infection	HIV RNA PCR positive No growth detected on bacterial, mycobacterial and fungal lymph node cultures
6	27	F	African American	Submental neck mass	Submental neck mass	None at presentation	-
7	34	M	-	-	-	-	-
8	28	F	Asian	Neck pain, headache, fever, night sweats and facial rash	Left supraclavicular	None at presentation	No growth detected on bacterial blood cultures or fungal, mycobacterial lymph node cultures

Abbreviations: F—female; HIV—human immunodeficiency virus; M—male; SLE—systemic lupus erythematosus; and IHC—immunohistochemistry.

## Data Availability

The original contributions presented in the study are included in the article, further inquiries can be directed to the corresponding author.
